# Lipid Profile Remodeling in Response to Nitrogen Deprivation in the Microalgae *Chlorella* sp. (Trebouxiophyceae) and *Nannochloropsis* sp. (Eustigmatophyceae)

**DOI:** 10.1371/journal.pone.0103389

**Published:** 2014-08-29

**Authors:** Gregory J. O. Martin, David R. A. Hill, Ian L. D. Olmstead, Amanda Bergamin, Melanie J. Shears, Daniel A. Dias, Sandra E. Kentish, Peter J. Scales, Cyrille Y. Botté, Damien L. Callahan

**Affiliations:** 1 Department of Chemical and Biomolecular Engineering, The University of Melbourne, Parkville, Victoria, Australia; 2 Metabolomics Australia, The School of Botany, The University of Melbourne, Parkville, Victoria, Australia; 3 Apicolipid Group, Laboratoire Adaption et Pathogenie des Microorganismes UMR5163, CNRS, University of Grenoble I, La Tronche, France; 4 Centre for Chemistry and Biotechnology, School of Life and Environmental Sciences, Deakin University, Burwood, Victoria, Australia; University of Geneva, Switzerland

## Abstract

Many species of microalgae produce greatly enhanced amounts of triacylglycerides (TAGs), the key product for biodiesel production, in response to specific environmental stresses. Improvement of TAG production by microalgae through optimization of growth regimes is of great interest. This relies on understanding microalgal lipid metabolism in relation to stress response in particular the deprivation of nutrients that can induce enhanced TAG synthesis. In this study, a detailed investigation of changes in lipid composition in *Chlorella* sp. and *Nannochloropsis* sp. in response to nitrogen deprivation (N-deprivation) was performed to provide novel mechanistic insights into the lipidome during stress. As expected, an increase in TAGs and an overall decrease in polar lipids were observed. However, while most membrane lipid classes (phosphoglycerolipids and glycolipids) were found to decrease, the non-nitrogen containing phosphatidylglycerol levels increased considerably in both algae from initially low levels. Of particular significance, it was observed that the acyl composition of TAGs in *Nannochloropsis* sp. remain relatively constant, whereas *Chlorella* sp. showed greater variability following N-deprivation. In both algae the overall fatty acid profiles of the polar lipid classes were largely unaffected by N-deprivation, suggesting a specific FA profile for each compartment is maintained to enable continued function despite considerable reductions in the amount of these lipids. The changes observed in the overall fatty acid profile were due primarily to the decrease in proportion of polar lipids to TAGs. This study provides the most detailed lipidomic information on two different microalgae with utility in biodiesel production and nutraceutical industries and proposes the mechanisms for this rearrangement. This research also highlights the usefulness of the latest MS-based approaches for microalgae lipid research.

## Introduction

Increasing and unstable oil prices, diminishing fossil fuel reserves, and increasing atmospheric CO_2_ levels have revitalized interest in microalgae as a renewable, lipid-rich feedstock for biofuel production [1]–[5]. Microalgae are attractive as feedstock for biofuel production primarily due to their ability to synthesize and accumulate high levels of TAGs in specialized lipid bodies located in the cytoplasm and to a lesser extent the chloroplast [6]. The fatty acids (FA) associated with the TAGs can be trans-esterified into fatty acid methyl esters (FAMEs) to produce biodiesel. The TAGs are the preferred source of FAs for biodiesel production as they can be converted to biodiesel using conventional methods [7], however, these acyl chains (used here interchangeably with FA) are mainly present as building blocks for the algal membrane lipids. These include major plastid and photosynthesis-related glycolipids such as monogalactosyldiacylglycerol (MGDG), digalactosyldiacylglycerol (DGDG) and sulfoquinovosyldiacylglycerol (SQDG); phosphoglycerolipids such as phosphatidylglycerol (PG), phosphatidylcholine (PC), phosphatidylethanolamine (PE), phosphatidylserine (PS) and phosphatidylinositol (PI), together with sphingolipids and other neutral lipids such as diacylglycerol (DAG). As the physical properties and quality of the biodiesel are directly dependent on the nature and chemical structures of the recovered FAs [8], [9], the relative amounts of each lipid class and their FA profiles largely determine the suitability of a given feedstock for biodiesel production.

The lipid composition of plant and algal cells are in a constant state of flux, with the relative amounts and localization of lipid species able to change in response to environmental conditions. The effect of changing conditions on total FA profile in microalgae has been reported, with studies showing that the FA profile can be affected by parameters such as the availability of phosphorus [10], nitrogen [11]–[13]; silicon [14]; temperature [15], [16]; salinity [17]; light level [18]; light/dark cycle [19], [20]; and growth phase of the culture [21], [22]. Changing environmental conditions have also been shown to affect the relative proportion of each lipid class in microalgae. Critically, certain types of stress, and in particular as N-deprivation, are known to increase the production of TAGs [23]. N-deprivation is seen as a practical means of enhancing biodiesel production from microalgae, and an increasingly detailed understanding of the underlying metabolism is required for its optimal implementation.

Our understanding of the regulation of lipid metabolism in photosynthetic organisms has been developing steadily over recent decades. Lipid metabolism in plants and algae involves a combination of *de novo* synthesis pathways, recycling pathways and lipid trafficking between sub-cellular compartments [24], [25]. These pathways must be tightly regulated to achieve lipid homeostasis and ensure the composition of each sub-cellular membrane is maintained or altered as appropriate for the environmental conditions. The available data on microalgae suggest that enhanced TAG production under N-deprivation results, at least in part, from the recycling of glycolipids combined with *de novo* synthesis pathways [25]–[28]. However, the precise nature of the perturbations to the lipidome that occur in microalgae under N-deprivation are yet to be determined.

To better understand the changes that occur in microalgae in response to N-deprivation, it is essential to obtain accurate and quantitative information about lipid composition. The identification and quantification of individual lipid species in biological samples has been greatly facilitated by recent advances in LC-MS and GC-MS [29]. These techniques have been successfully employed for the analysis of plant lipids [30], [31], including under normal versus physiologically-stressed conditions [32], and enabled the characterization of MGDG and DGDG in photosynthetic marine protists [33], [34], and SQDG [35], betaine lipids [16] and TAGs [14], [36] in various species of algae.

In this work, we use LC-MS and GC-MS to obtain detailed lipidomic information for two model microalgal species from the chromophyte (golden-brown) and chlorophyte (green) lineages under N-replete and N-deplete conditions. These data provide important new information on the effect of N-deprivation on FA profiles across several key lipid classes, and in particular within TAGs, which is discussed in the context of current knowledge of the metabolic responses of microalgae to nutrient stress.

## Materials and Methods

### 2.1 Algal strains

Cultures of Chlorella sp. and Nannochloropsis sp. were maintained in 25 mL clear plastic tissue culture flasks at 17°C in a light∶dark cycle of 12∶12 hr with a photon flux density of 48–55 µE.m^−2^.s^−1^ in a marine medium as previously described [37]. For the experimental work, 1.2 L of aerated cultures of the two algal species were grown in aerated 2 L Schott bottles at 17°C with a photon flux density of 60–70 µE.m^−2^.s^−1^. The aeration provided both a source of carbon and agitation for the algal cells in culture. Nitrogen-replete (control) cultures were established with 5 mM NO_3_^−^, at this concentration nitrogen was not growth-limiting within the time of the experiment. N-deplete cultures were established in fresh medium which contained 0.5 mM NO_3_^−^ for both species. These cultures were allowed to grow for 7–10 days, then harvested by continuous centrifugation (5000 g) and immediate lipid extraction.

### 2.2 Lipid extraction

Lipids were extracted using a modified Bligh and Dyer [38] procedure as previously described [37]. The extracts were dried under nitrogen, weighed and stored under nitrogen at −20°C until used for the LC-MS analysis.

### 2.3 LC-MS

The dried algal lipid extracts were re-suspended in butanol/methanol (1∶1, v/v) containing 10 mM butylated hydoxy toluene (BHT) for analysis by LC-MS. The BHT was added to improve the oxidative stability of extracts. An Agilent 1200 series LC system equipped with a vacuum degasser, binary pump, temperature controlled autosampler and column oven was used for chromatography. Lipids were fractionated using an Ascentis Express 50 mm×2.1 mm, 2.7 µm particle size RP amide column (Supelco-SigmaAldrich) which was maintained at 35°C. Lipids were eluted using a binary mobile phase gradient at a flow rate of 0.2 mL·min^−1^. Mobile phase (A) comprised water/methanol/tetrahydrofuran (50∶20∶30, v/v/v) with 10 mM ammonium formate, mobile phase (B) comprised water/methanol/tetrahydrofuran (5∶20∶75, v/v/v) with 10 mM ammonium formate. The gradient started at 100% A and linearly decreased to 0% A over 10 min with a one min hold at 0% A, then re-equilibration at 100% A for 4 minutes. Lipids were measured using an Agilent 6410b electrospray ionization-triple quadrupole (ESI-QQQ)-MS.

Multiple reaction monitoring (MRM) lists for phospholipids were developed for quantification by initial untargeted scans using the precursor and neutral loss scan functions. The following lipids were analysed using precursor ion scanning in positive ion mode: PC (precursors of m/z 184.1), cholesterol esters (m/z 369.4), PG (m/z 189) and in negative ion mode: PI (m/z 241), phosphatidic acid PA (m/z 153) and SQDG (m/z 225). Positive ion neutral loss scanning was used to identify PE (neutral loss of 141 u), MGDG (neutral loss of 179 u), DGDG (neutral loss of 341 u) and in negative ion neutral loss mode: PS (neutral loss of 87 u). The neutral lipids were analysed in single ion monitoring mode (SIM). The SIM targets for mono-, di- and triacylglycerols were created using all combinations of the identified FAs from the total GC-MS data [37]. A maximum of 100 MRMs or SIMs (5 ms dwell times) were analysed per time segment providing approximately 12–16 data points across a chromatographic peak. Optimized parameters for capillary, fragmentor, and collision voltages were 4,000, 60–160, and 0–60 V, respectively (details in Table S1).

Single ion monitoring was used to quantify neutral lipids due to the different response factors which arise from fragmentation of DAGs and TAGs. The predominant product ions arising from MS/MS of DAGs and TAGs are from the neutral loss of fatty acids. The product ions produced from TAG's with three different fatty acids do not necessarily produce a 1∶1∶1 ratio from the neutral loss of each fatty acid. Also, the fragmentation of a TAG with the same fatty acids in all three SN positions also produces a higher response factor when compared with a TAG with a mix of fatty acids. This means that a single MRM cannot be selected for TAGs and DAGs when using a single representative standard, in this case triolein (TAG18∶1/18∶1/18∶1) and diolein (DAG18∶1/18∶1) and therefore single ion monitoring was used for DAGs and TAGs. This acquisition approach measures the abundance of intact lipids species.

The data were processed using Agilent Mass Hunter Qualitative (for scan data) and Quantitative (for MRM and SIM data) analysis software. A combined 50 µM external calibration standard containing deuterated phospholipids PC(16∶0/18∶1)d31 (5 isotopomers), PE(16∶0/18∶1)d31, PG(16∶0/18∶1)d31 (3-isotopomers present), PA(16∶0/18∶1)d31, PS(16∶0/18∶1)d31, PI(16∶0/18∶1)d31 and glycolipids MGDG(34∶6/36∶6), DGDG(34∶6/36∶6), SQDG(34∶1) was prepared. A second combined external standard (100 µM) of mono, di- and triacylglycerol standard (MAG-18∶1; DAG-18∶1/18∶1; TAG-18∶1/18∶1/18∶1; Nu-Chek Prep >98%) was also prepared. A single point calibration was used for quantification for each lipid against the standard from the same class and the final concentrations calculated using the extracted lipid mass for each algal extract. Where more than one isotopomer or lipid was present (e.g. PE the sum of the areas for the multiple peaks in the standard was used for quantification. Detected lipid species were annotated as follows; lipid class (sum of carbon atoms in the two fatty acid chains∶sum of double bonds in the fatty acid chains).

## Results and Discussion

**Figure 1:**
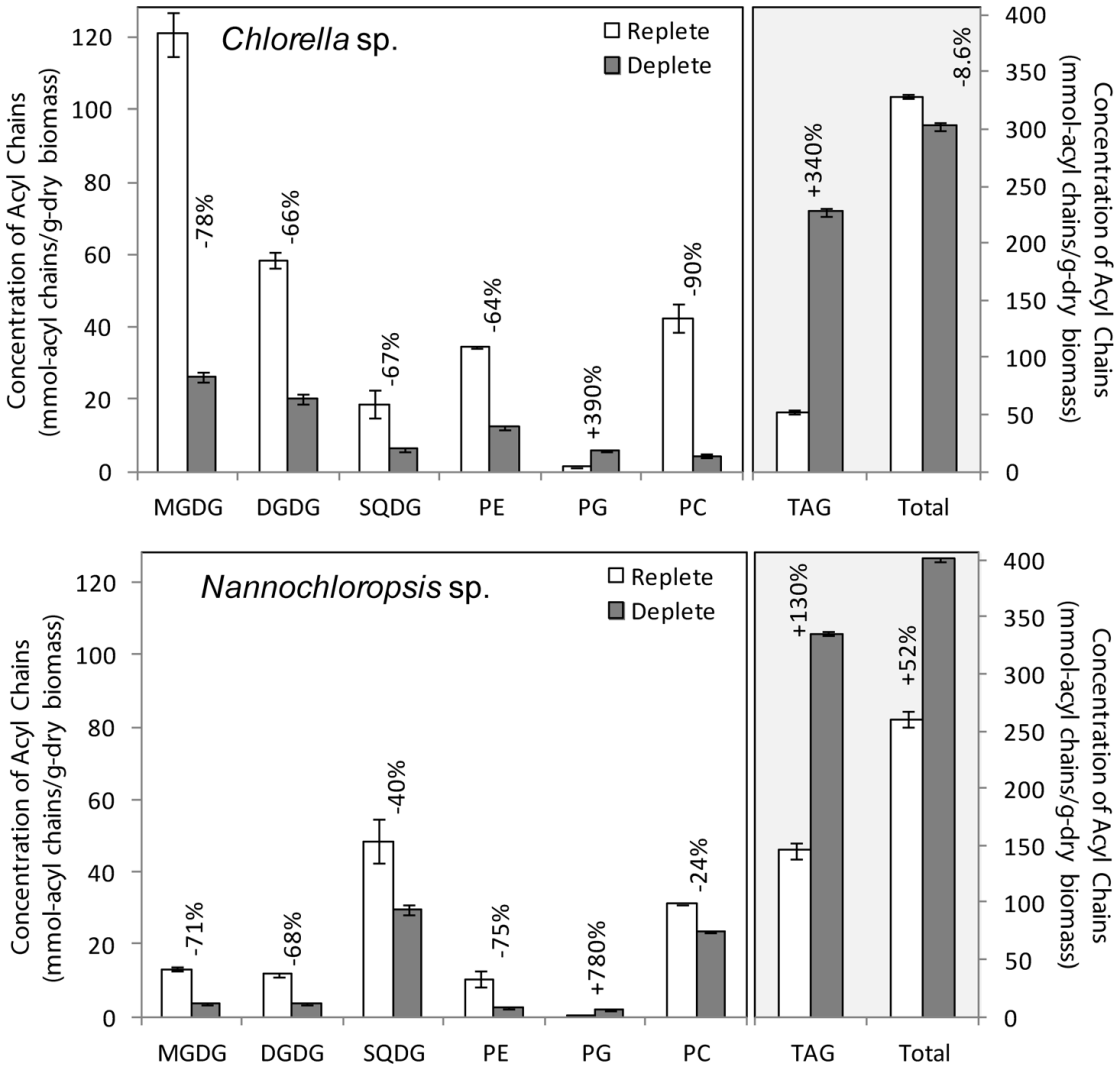
Total acyl chain concentrations within different lipid classes recovered from nitrogen replete and deplete *Chlorella* sp. and *Nannochloropsis* sp. The TAGs and total lipids read from right hand axis, all other lipid classes read from the left hand axis. Error bars represent the standard deviation of triplicate experiments.

**Figure 2:**
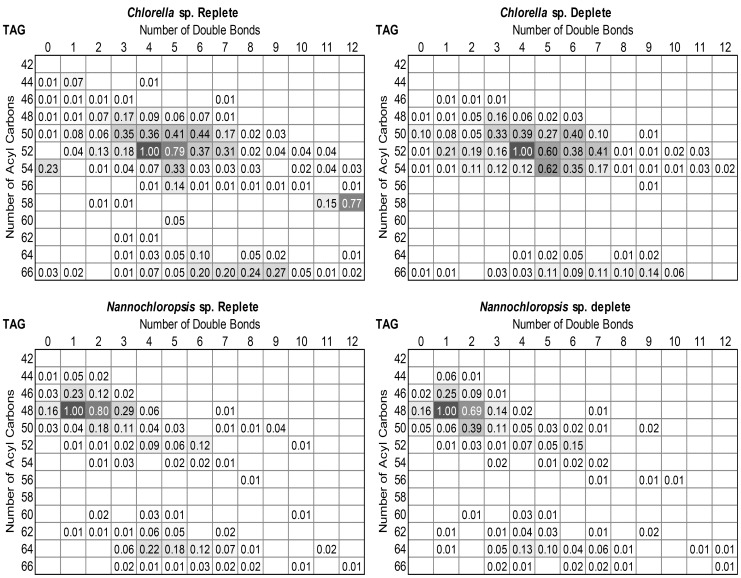
Heat mapped profile of combined acyl content (3 acyl chains) of TAG in nitrogen replete and nitrogen deplete *Chlorella* sp. and *Nannochloropsis* sp. The assigned values represent the relative proportion of each species normalised to the most prevalent species in each (e.g. 52∶4 for nitrogen replete *Chlorella* sp.). Values less than 0.01 are not presented.

**Figure 3:**
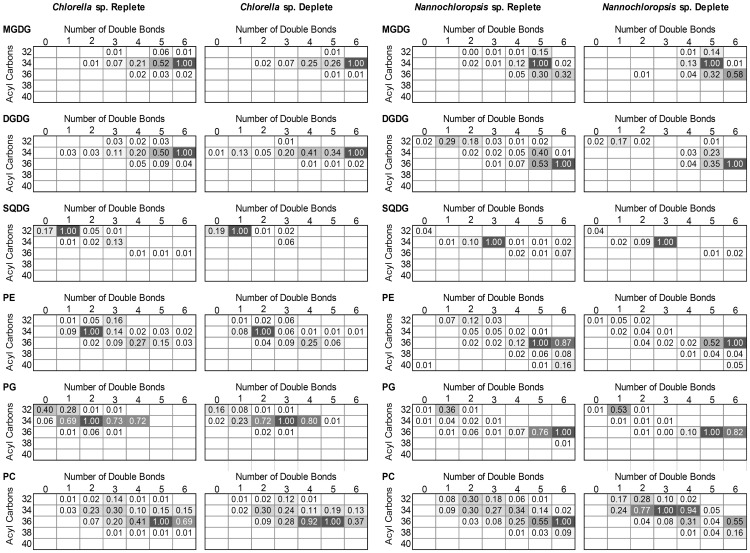
Heat mapped profiles of combined acyl content (2 acyl chains) of glyco- and phosphoglycerolipids in nitrogen replete and nitrogen deplete *Chlorella* sp. and *Nannochloropsis* sp. The assigned values represent the relative proportion of each species normalised to the most prevalent species in each. Values less than 0.01 are not presented.

**Figure 4:**
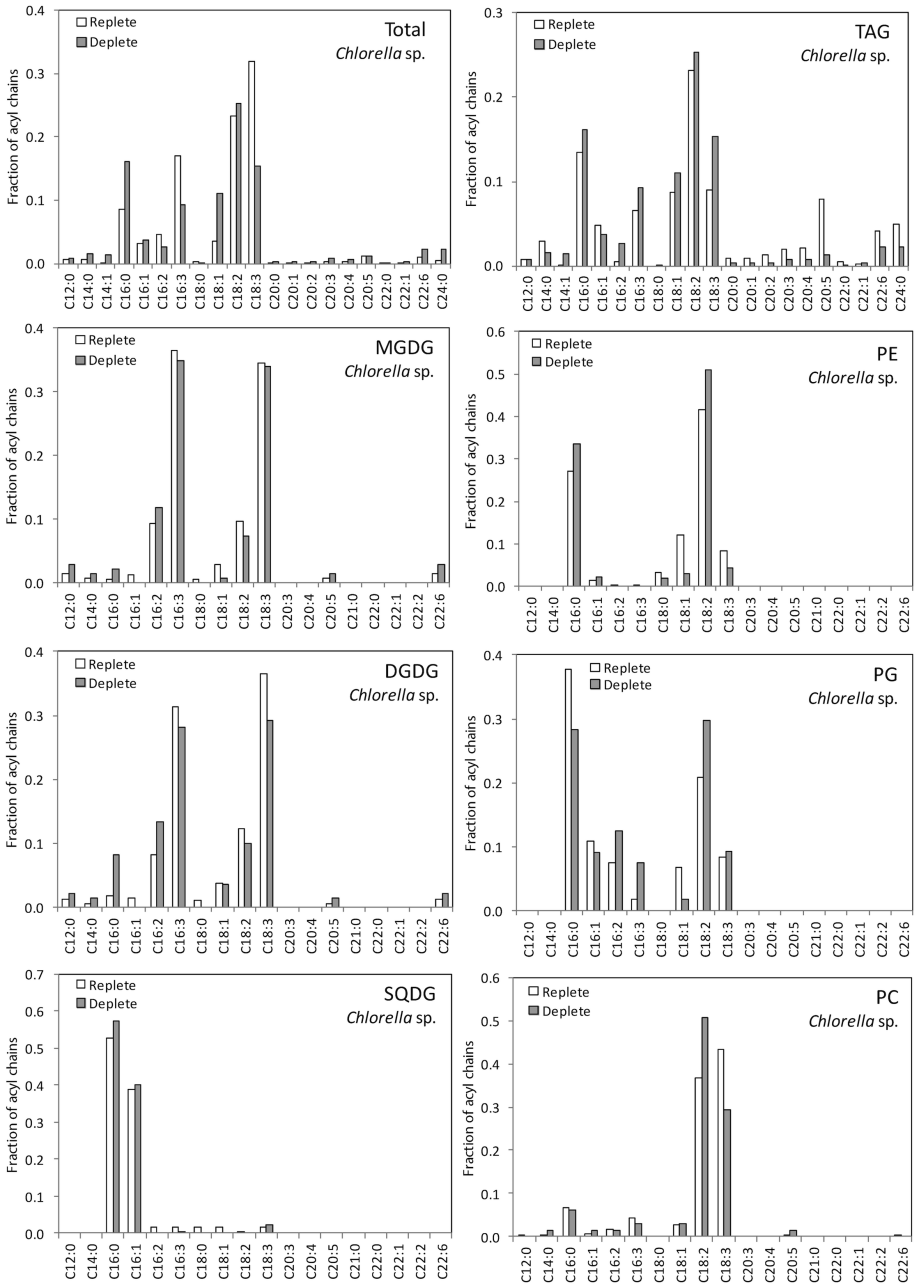
Estimated acyl chain composition as a function of lipid class based on combined LC-MS and lipid class fractionated GC-MS data [37] for nitrogen replete and deplete *Chlorella* sp.

**Figure 5:**
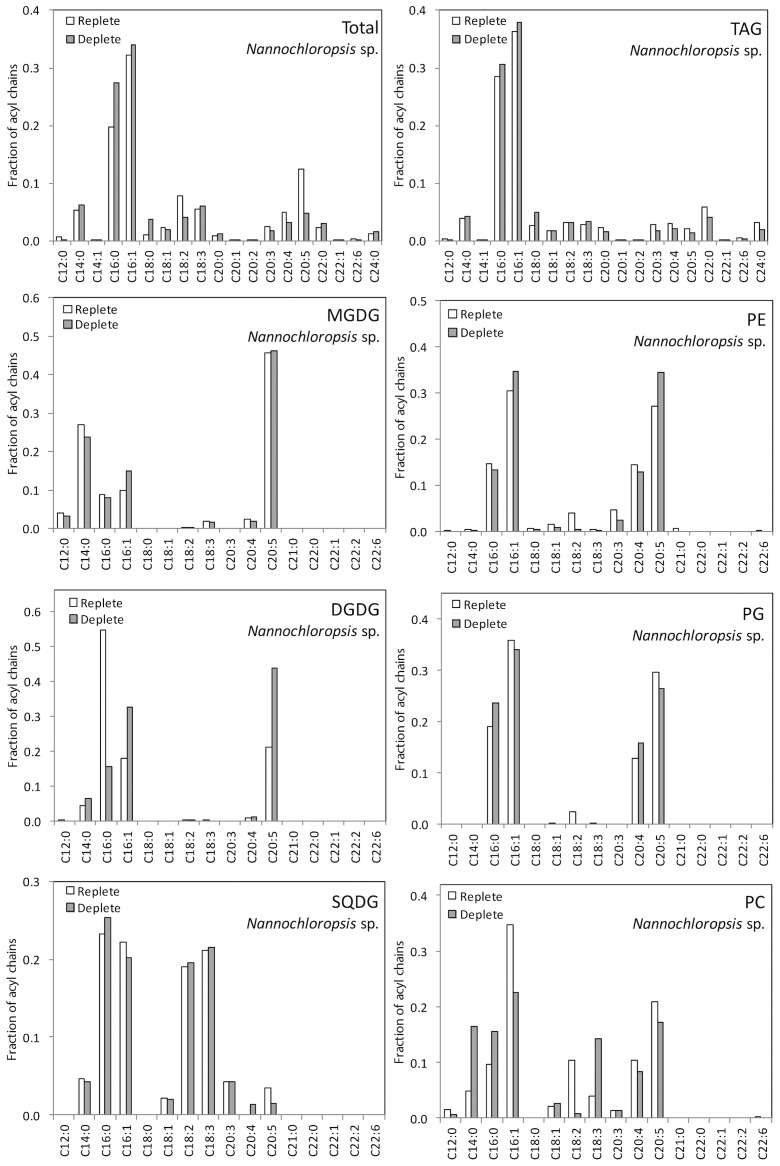
Estimated acyl chain composition as a function of lipid class based on combined LC-MS and lipid class fractionated GC-MS data [37] for nitrogen replete and deplete *Nannochloropsis* sp.

The LC-MS data provided quantitative distributions of key lipid classes on the basis of total acyl chain concentration in *Chlorella* sp. and *Nannochloropsis* sp. under N-replete and N-deplete conditions (Figure 1) and profiles of the combined acyl content of each lipid class (Figures 2 and 3). The LC-MS approach used here provided the combined mass of the acyl chains attached to a particular head group (see Table S2 for complete data set). As it is possible for lipids with multiple acyl chains to have different combinations of FAs sharing the same overall mass, the individual acyl content was not determined directly. While an MS/MS analysis on each lipid could determine the exact FA composition this was not practical for the extensive number of lipids examined in this work. The assignment of fatty acid compositions for the glycolipids was ambiguous therefore MS/MS of the high abundant MGDG and DGDG lipids was carried out to confirm the FA composition (Figure S1) For the other classes the LC-MS data was combined with previously obtained GC-MS data for these samples [37], allowing the most probable combination of individual fatty acids to each lipid species to be ascertained (Figures 4 and 5, and Table S3). The compatibility and consistency of the two data sets was cross-checked by comparing the FA profile of the overall lipid content, TAGs (neutral lipids), and polar lipids (glycolipids and phospholipids) as determined by the two different methods (Table S4). Considering the fundamental differences between these two methods, the two data sets were in good agreement.

### 3.1 Quantitative distribution of lipid classes before and after N-deprivation

In agreement with previous studies [12], [28], [37], [39]–[41] N-deprivation led to a considerable accumulation of TAGs in both species (Figure 1), increasing by 340% in *Chlorella* sp. and 130% in *Nannochloropsis* sp. Despite the differences in percentage increase, however, after N-deprivation the total amount of TAGs was still higher in *Nannochloropsis* sp. at 336±2 mmol_acyl chains_/g_dry biomass_ compared to 228±3 mmol_acyl chains_/g_dry biomass_ in *Chlorella* sp.

In both species, the increase in TAG content was associated with a decrease in total phosphoglycerolipid content, on a dry biomass basis (Figure 1). The amount of phosphoglycerolipid by weight decreased from 78±4 to 23±1 mmol_acyl chains_/g_dry biomass_ in *Chlorella* sp. and from 42±2 to 28±1 mmol_acyl chains_/g_dry biomass_ in *Nannochloropsis* sp. Analysis of individual phosphoglycerolipid classes revealed that following N-deprivation, PE decreased by 64–75% and PC decreased by 24–90%, while the levels of PS, PI and PA remained below the threshold of detection. As both PE and PC contain nitrogen in their head group, these decreases most likely reflect reduced capacity to synthesize these species following N-deprivation. By contrast, N-deprivation resulted in elevated levels of PG in both species, increasing by 390% in *Chlorella* sp. and 780% in *Nannochloropsis* sp. Despite these large percentage increases, however, the PG content by weight remained relatively low in both species.

The increase in TAGs following N-deprivation was similarly associated with a decrease in total glycolipid content. The amount of glycolipid by weight decreased from 200±12 to 52±3 mmol_acyl chains_/g_dry_ _biomass_ in *Chlorella* sp. and from 73±7 to 37±2 mmol_acyl chains_/g_dry biomass_ in *Nannochloropsis* sp. Analysis of individual glycolipid classes revealed that N-deprivation led to decreases in MGDG and DGDG of 66–78% and a decrease SQDG of 40–67%. Interestingly, the most abundant glycolipid species differed between the two algae. In *Chlorella* sp., MGDG was the most abundant of the chloroplast glycolipids, consistent with observations in a variety of photosynthetic organisms [42]. In *Nannochloropsis* sp., SQDG was the most abundant chloroplast glycolipid, a feature shared with only a few photosynthetic bacteria and algae.

Data from studies in other photosynthetic organisms suggest the increase in PG and the decrease in chloroplast glycolipids are likely to be related. Phosphatidylglycerol is the only phosphoglycerolipid that is found in the photosynthetic membranes of plastids [42], where it is necessary for the functions of photosystems I and II and the light harvesting complex II [43]. Photosynthesis and chloroplast development are also known to be dependent on both MGDG and DGDG [33], [44], and SQDG [43]. The significant increase in PG with N-deprivation may therefore be to compensate for the loss of chloroplast glycolipids in an attempt to maintain proper photosynthetic activity. Alternatively, as N-deprivation has been shown to produce algal cells with smaller chloroplasts containing fewer thylakoids (photosynthetic membranes), less pigment and a disruption to photosynthetic capacity along with a reduction in the level of proteins involved in the photosynthetic electron transport chain [28], [45], the decrease in glycolipids observed could also be due to a reduction in chloroplast size or thylakoid number. Another contributing factor to the observed decrease in membrane lipids following N-deprivation is the increase in average cell size that also results from generation of large TAGs-containing lipid bodies (or lipid droplets). If the data were obtained on a per cell basis rather than on a dry biomass basis as presented here, the apparent reduction in membrane lipids would be less and the increase in TAGs would be greater.

### 3.2 Comparison of acyl content of different lipid classes

The LC-MS was used to identify and quantify individual lipid species of a particular mass within each of the major lipid classes (Figures 2 and 3). The most prominent TAG species in *Nannochloropsis* sp. under both N-replete and N-deplete conditions were 48∶1 and 48∶2 (Figure 2), which likely consist of 16∶0 and 16∶1 acyl chains (Table S3). The TAG profile in *Nannochloropsis* sp. shows only a few changes in response to N-deprivation. The most notable change is the increase in the levels of 50∶2 (likely comprising 18∶1, 16∶1 and 16∶0). As the *de novo* production of these FA species has previously been observed with nutrient deprivation e.g. [46], the increase in the 50∶2 species suggests a similar response occurred here upon N-deprivation. There was also a notable decrease in the 64∶4 and 64∶5 species in *Nannochloropsis* sp. following N-depletion (Figure 2), indicating long polyunsaturated FA species (e.g. 20∶4, 20∶5) did not accumulate in the TAGs. These long FA chains and more particularly C20∶5 (eicosapentaenoic acid or EPA) are usually found in the chloroplast galactolipids MGDG and DGDG (Figure 5). Here EPA was found be globally decreased in PG, SQDG and PC but was found in higher abundance in DGDG and PE under nitrogen deprivation. These observations suggest that EPA could be exported outside the chloroplast via DGDG or free FA to be eventually re-utilised for purposes other than TAG synthesis. Since EPA is a good source of reducing power, it could be recycled by the cell as an easy energy source during nitrogen-stress. Future experiments could confirm this by measuring the activity of beta oxidation and/or reducing power stocks under stress. There was a slight increase in 48∶1 relative to 48∶2 following N-depletion (Figure 2), reflecting the increase in 16∶0 compared to 16∶1 observed in GC-MS analysis (Figure 5). This suggests that newly synthesized FAs could be directly incorporated into TAGs prior to their desaturation, potentially as an energy saving mechanism.

The profile of TAGs in *Chlorella* sp. was more diverse than in *Nannochloropsis* sp. The most abundant species were 50∶4/5, 52∶3/4/5/6, 54∶0/3, 58∶12 and 66∶5–10 (Figure 2, Table S2). In comparison to *Nannochloropsis* sp., the TAG content of *Chlorella* sp. showed more variability upon N-deprivation, indicating that more FA exchange and trafficking could occur during the stress (Figure 2). Of significance was the near disappearance of 58∶12 (comprising 20∶5, 20∶5 and 18∶2). This disappearance was associated with an increase in TAG species possessing a single 20∶5 in combination with shorter and more saturated FAs such as 52∶7 (20∶5/16∶1/16∶1) and 54∶5 (20∶5/18∶0/16∶0). There was also a clear increase of 54∶2–7 species, suggesting TAGs were enriched in C18∶0/C18∶1/C18∶2 (Figure 2, Table S4). The stable levels of 20∶5 in TAGs under N-deprivation suggest that 20∶5 was being recycled by the cell. Usually, EPA (20∶5) is associated with chloroplast lipids as observed here (Figure 4), although not as their major components as detected in *Nannochloropsis* sp. (Figure 5). Furthermore, since none of lipid classes of *Chlorella* sp. showed any significant decrease in EPA, it could indicate that this FA species is mainly recycled directly from existing TAGs and replaced by other FA species in the TAG pool during nitrogen deprivation. The reason for recycling or specific replacement of EPA remains to be elucidated. A noticeable change in TAG content is the increase of C16∶3, C18∶3 and C18∶2. C18∶3 and C16∶3 are typically found in chloroplast membranes (Figure 4). Both species were found to significantly decrease in DGDG and at a lesser extent, in MGDG, both major chloroplasts lipids species. A possible explanation for the C16∶3/C18∶3-containing TAG population is the direct recycling of chloroplast galactolipids. The cell could eventually compensate its loss of chloroplast fatty acid by increasing *de novo* fatty acid production under stress conditions, as suggested by (i) an increase of mid-long fatty acid chains C16∶0/C18∶0/C18∶1/C18∶2 in MGDG, DGDG (and SQDG), (ii) an increase of C16∶3/C18∶3 in less abundant chloroplast classes PG and SQDG and (iii) a global increase of thylakoids/photosystems-interacting lipid class PG (Figure 5).There was also a decrease of very long chain-containing TAGs (66∶6–9), without a significant decrease of 22∶6 or 20∶4 in other lipid species (Figure 4) indicating these TAGs were likely utilized for other purposes (such as beta-oxidation) rather than being remodelled. Finally, levels of 58∶1–3 were also higher in N-deprived conditions (16∶0, 18∶1 and 18∶2), again likely representing an increase in *de novo* FA synthesis in TAGs. The 16∶0 and 18∶1 are normally the newly synthesized FA species, so their increase in TAGs suggests that on top of recycling “mature” lipids for TAG generation, the cell is also actively using its *de novo* synthesis pathways, and potentially recycling lipids for other purposes.

Other changes could be detected in glycerophospholipids during N-deprivation, each specific to FA abundances/utilisation depending on the microalgal species. Indeed, the PC, PE and PG of *Chlorella* sp. were all subjected to an increase in C18∶2 content, whilst containing less C18∶3 (with the exception of PG for the latter). This increase in C18∶2 was also detected in the TAG profile (Figure 4) concomitant with the increase of C18∶3. On the other hand, the glycerophospholipids of *Nannochloropsis* sp. showed different behaviour. Here the main changes occurred at the level of C14∶0, C16∶0/1 and C20∶4/5 with shorter and less unsaturated chains found to increase in PC, PE and PG, whilst EPA was reduced in PC and PG.

One key difference in the overall FA profiles of the two species (compare Figures 4 and 5) is that *Chlorella* sp. has a preference for the production of the prokaryotic- and eukaryotic-generated C16∶0 and C18∶3 typically found in chloroplast glycolipids in higher plants whereas *Nannochloropsis* sp. produces more EPA (20∶5). The significance of this distribution is unclear, but follows a general rule that chromist (golden-brown) algae (including *Nannochloropsis*) produce EPA while chlorophyte (green) algae (including *Chlorella*), if any, produce DHA.

### 3.3 Interpretation of biosynthetic rearrangements due to nitrogen deprivation

Our data shows N-deprivation leads to a considerable increase in TAG and PG content and a concomitant decrease in polar lipid content in both species of microalgae. Despite these changes, however, the FA profiles within each lipid class remained relatively constant. As previously mentioned, phosphoglycerolipids are important structural components of extra-plastidial membrane. The maintenance of their FA profiles therefore shows the composition of extra-plastidial membranes is not significantly altered by N-deprivation. Conversely, the glycolipids which are key components of chloroplast membranes are required to support photosynthesis and although their FA and species profiles were also largely maintained, the increase in PG content suggests that this alone was insufficient for optimal photosynthetic activity.

**Figure 6:**
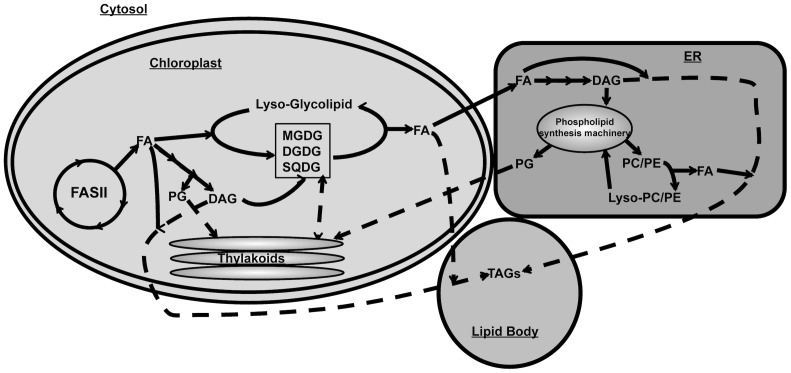
Proposed pathways for TAG synthesis during N-starvation.

We therefore propose that N-depletion triggers major remodelling of intracellular lipid pools in both *Chlorella* sp. and *Nannochloropsis* sp. in order to boost TAG synthesis in lipid bodies whilst maintaining lipid homeostasis in other compartments (Figure 6). Even though the two species responded differently, the major trends appear to be conserved. In both *Chlorella* sp. and *Nannochloropsis* sp., the chloroplast glycolipids appear to be broken down as previously proposed [26]–[28], [45], that are then recycled for TAG synthesis. *Nannochloropsis spp.* seemed to incorporate shorter and more saturated FA (C16∶0, C16∶1) from neosynthesized galactolipids, whereas *Chlorella spp.* used a combination of C18∶2 and C18∶3 species, likely originating from mature galactolipids, and likely neosynthesized C18∶0/C18∶1. Interestingly, both species contained EPA (C20∶5) in TAGs or galactolipids prior to nitrogen stress but this FA specie did not increase and/or decreased in TAGs species and other glycerolipids upon N-deprivation, suggesting it was either metabolized (e.g. by beta-oxidation) or modified for a yet to be determined reason.

An enzyme capable of cleaving FAs from MGDG has been identified in *Chlamydomonas reinhardtii* [45] providing a possible mechanism for recycling of this glycolipid. Based on our data, we would suggest that similar enzymes also exist for the liberation of FAs from DGDG and SQDG. To compensate for the loss of the chloroplast glycolipids, both algae appear to up-regulate PG synthesis. In both cases, the significant increases in PG levels were concomitant to the loss of SQDG, the other acidic lipid class found in the plastid. Thus, FAs derived from the breakdown of SQDG could be used in TAG synthesis while PG replaced SQDG in the maintenance of chloroplast function and structural integrity. Indeed, an increase in PG has been observed in SQDG-deficient mutant and sulphur-starved *Chlamydomonas*, suggesting the replacement of SQDG with PG may be common to both stress responses [47]. At the same time, additional FAs for TAG synthesis appear to be generated by the chloroplast *de novo* FA synthesis pathway [48]. These FAs may be exported to the lipid body directly, or may transit indirectly after first being incorporated into DAG in the endoplasmic reticulum (ER) or chloroplast (Figure 6). Finally, the breakdown and recycling of existing phosphoglycerolipids in the ER is likely to provide yet another source of FAs for TAG synthesis during N-deprivation.

## Conclusions

This work provides the most detailed analysis of the microalgal lipidome after nitrogen deprivation using new LC-MS approach. These data showed the expected increase in the concentrations of TAGs with a concomitant decrease in the levels of the chloroplast glycolipids, MGDG, DGDG and SQDG, as well as two major nitrogen containing phosphoglycerolipids, PC and PE after N-deprivation. In contrast, PG levels were highly increased in both models (from very low starting levels), a previously unnoticed effect of N-deprivation. Some modifications of FAs derived from the breakdown of existing glycolipids and phosphoglycerolipids in *Nannochloropsis spp.* and long FA chains from TAGs in *Chlorella* sp. occurs during nitrogen deprivation. This finding has great implications for the nutraceutical and biodiesel industry. It also shows that major remodelling of the intracellular lipid pools in both species occurs in order to boost TAG synthesis whilst attempting to maintain lipid homeostasis in other essential compartments such as the chloroplast.

The detailed quantitative information provided by the application of LC-MS illustrates the utility of this technique for studying lipid biochemistry and metabolism in microalgae and for the development of biotechnological applications such as biodiesel production. Liquid chromatography-MS provides the most comprehensive coverage of lipids with minimal sample handling. This enables investigations to be performed using small scale cultures, facilitating more extensive investigations across a range of growth variables and species.

## Supporting Information

Figure S1 **Example LC-MS/MS spectra of high abundant glycolipids in** ***Nannochloropsis*** **sp. and** ***Chlorella*** **sp.** Fatty acid assignments are included on the spectra. All fragment ions listed show the fatty acid chain length and degree of saturation for an ion of the following structure: [MAG(FA)-H_2_O+H^+^]^+^. Note: Gal = neutral loss of galactose.
(DOCX)

Table S1 **Collision energy, fragmentor voltage and ionisation polarity settings used for the LC-QQQ-MS analysis of each lipid class.** The ESI source settings were the same across all lipid classes.
(DOCX)

Table S2 **Complete LC-MS data set for the TAG, MGDG, DGDG, SQDG, PE, PG, and PC lipids.** Data represent the average and standard deviation of triplicate experiments and are presented in units of mmol-lipid/g-dry biomass.
(XLS)

Table S3 **Assignment of acyl chains to individual lipid species determined by manual matching of LC-MS and GC-MS data (from Olmstead et al. 2013).** Acyl chains were assigned to: LC-MS TAG data based on GC-MS data of the neutral lipid SPE fraction; LC-MS data of the combined pool of MGDG, DGDG and SQDG based on GC-MS data of the glycolipid SPE fraction; LC-MS data of the combined pool of PE, PG, PC based on GC-MS data of the phospholipid SPE fraction.
(XLS)

Table S4 **Heat map comparison of relative acyl chain abundance in the total lipids, TAGs, and polar lipids (glycol- plus phospholipids) of nutrient replete and deplete** ***Chlorella*** **sp. and** ***Nannochloropsis*** **sp. as determined by direct GC-MS analysis and inferred from LC data cross checked with GC results.** The assigned values represent the fraction of the total acyl in each lipid group represented by a particular fatty acid species. The GC-MS data for C16∶2 and C16∶3 acyl groups were quantified assuming consistent detector responses with the C16∶1 peaks, as no standards were available.
(DOCX)
