# Supplementary material for: Lipid Profile Remodeling in Response to Nitrogen Deprivation in the Microalgae Chlorella sp. (Trebouxiophyceae) and Nannochloropsis sp. (Eustigmatophyceae)
Source: PLoS One. 2014 Aug 29;9(8):e103389. doi: 10.1371/journal.pone.0103389 (PMC4149361; doi:10.1371/journal.pone.0103389)
Supplement: Table S1 — Collision energy, fragmentor voltage and ionisation polarity settings used for the LC-QQQ-MS analysis of each lipid class. The ESI source settings were the same across all lipid classes. (DOCX) [file pone.0103389.s002.docx]

| **Lipid class** | **Collision energy (V)** | **Fragmentor voltage (V)** | **Polarity** |
| --- | --- | --- | --- |
| PI | 60 | 160 | Negative |
| PA | 45 | 150 | Negative |
| PS | 20 | 160 | Negative |
| SQDG | 20 | 135 | Negative |
| PE | 20 | 160 | Positive |
| PG | 20 | 120 | Positive |
| PC | 30 | 60 | Positive |
| MGDG | 20 | 135 | Positive |
| DGDG | 20 | 135 | Positive |
| DAG | 0 | 135 | Positive |
| TAG | 0 | 135 | Positive |
