# Supplementary material for: Lipid Profile Remodeling in Response to Nitrogen Deprivation in the Microalgae Chlorella sp. (Trebouxiophyceae) and Nannochloropsis sp. (Eustigmatophyceae)
Source: PLoS One. 2014 Aug 29;9(8):e103389. doi: 10.1371/journal.pone.0103389 (PMC4149361; doi:10.1371/journal.pone.0103389)

Nannochloropsis replete

MGDG(32:5)

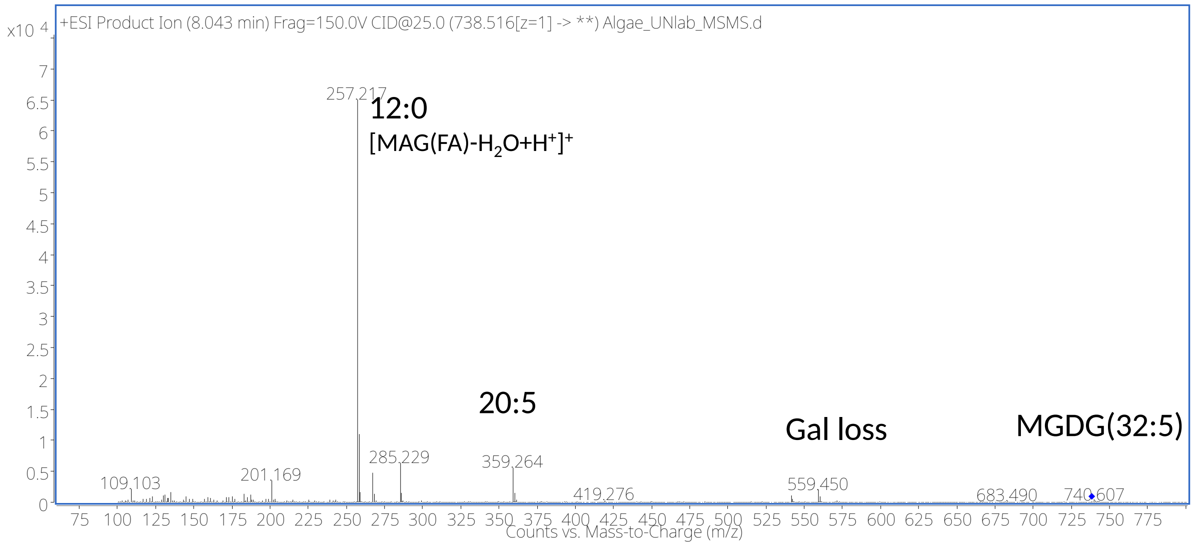

All fragment ions listed as numbers show the fatty acid chain length and saturation for an ion of the following structure: [MAG(FA)-H_2O+H^+]$^+$. Note Gal = neutral loss of galactose.

Nannochloropsis replete

MGDG(34:5)

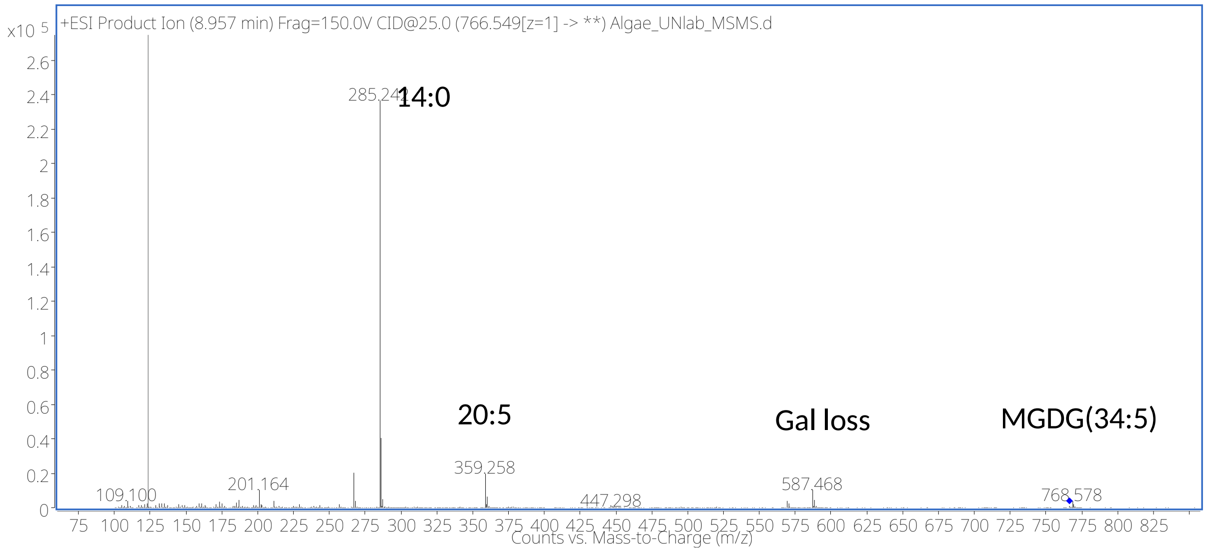

Nannochloropsis replete

MGDG(36:5)

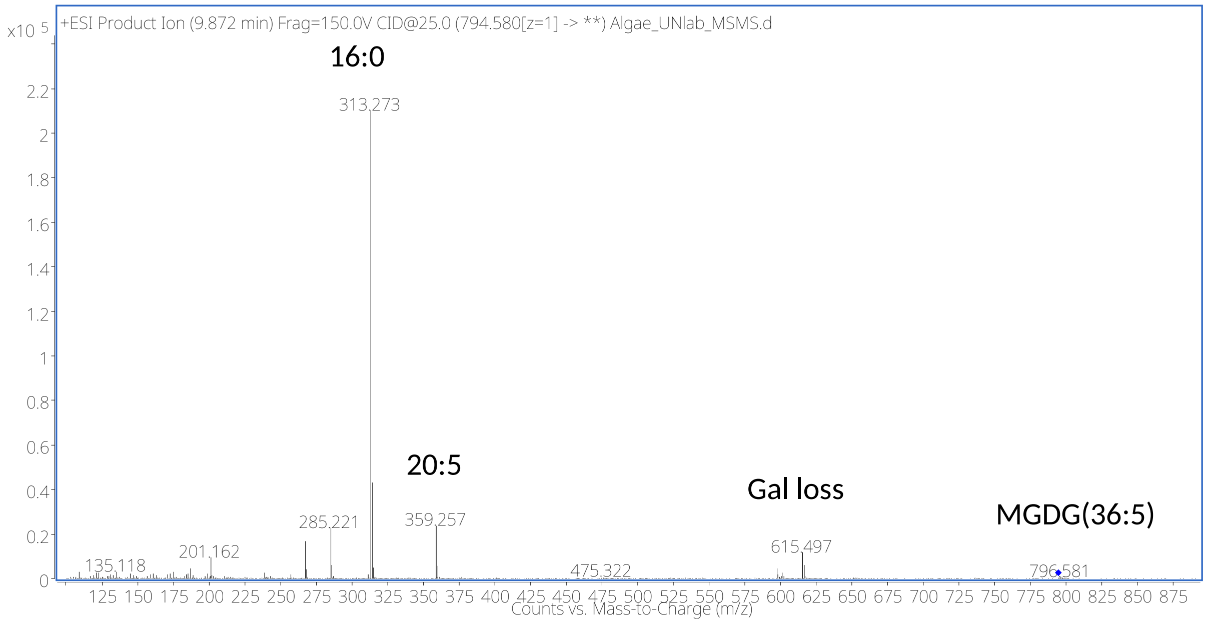

***Nannochloropsis* replete**

MGDG(36:6)

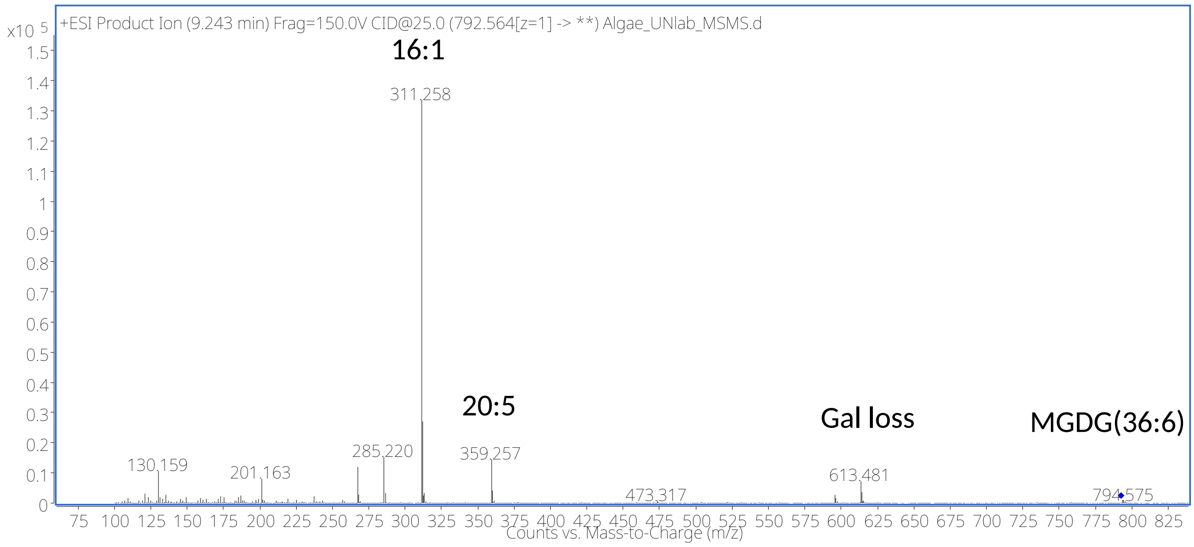

***Chlorella* replete**

Note: compare with Nannochloropsis

MGDG(32:5)

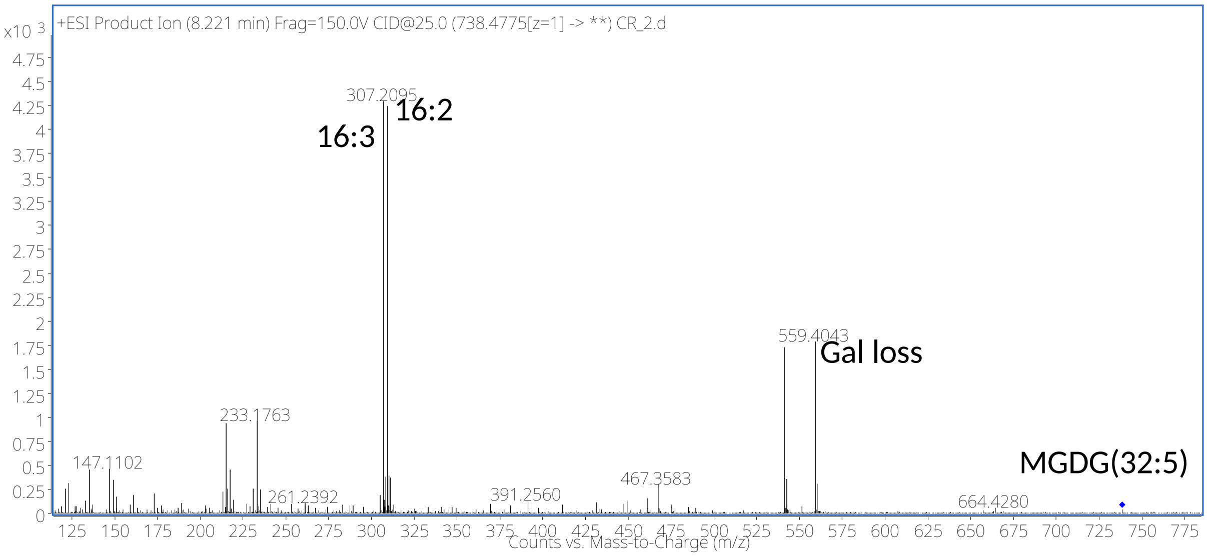

***Chlorella* replete**

MGDG(34:5)

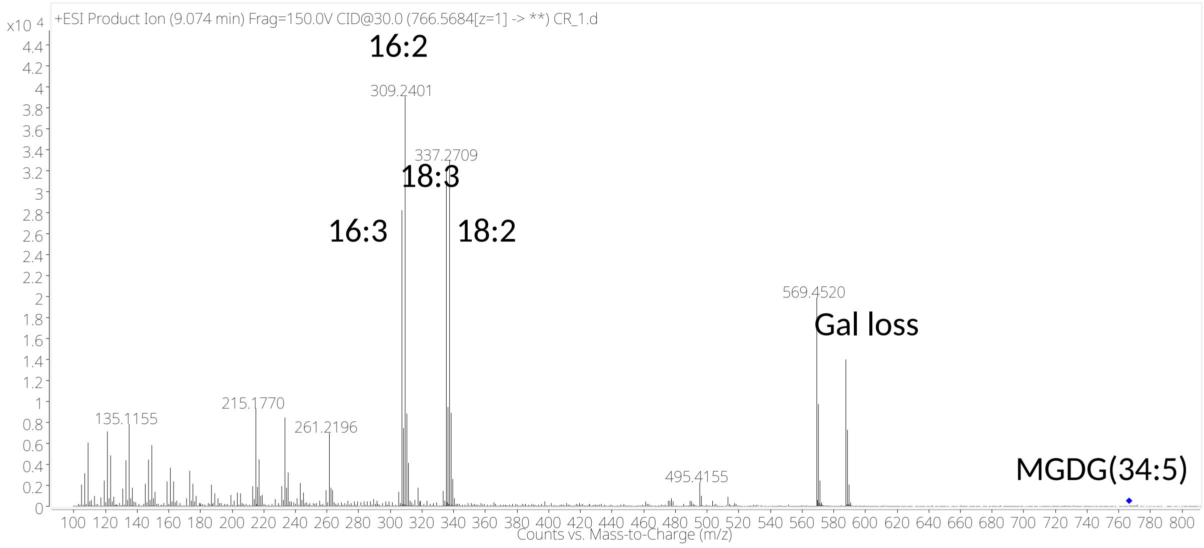

***Chlorella* replete**

MGDG(34:4)

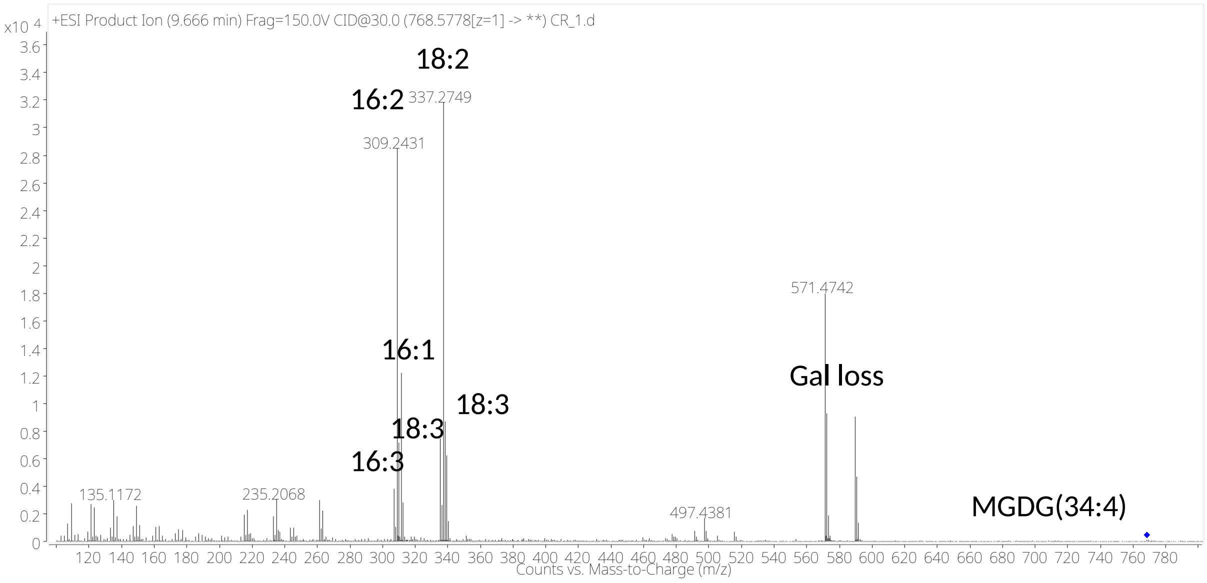

***Chlorella* replete**

MGDG(34:6)

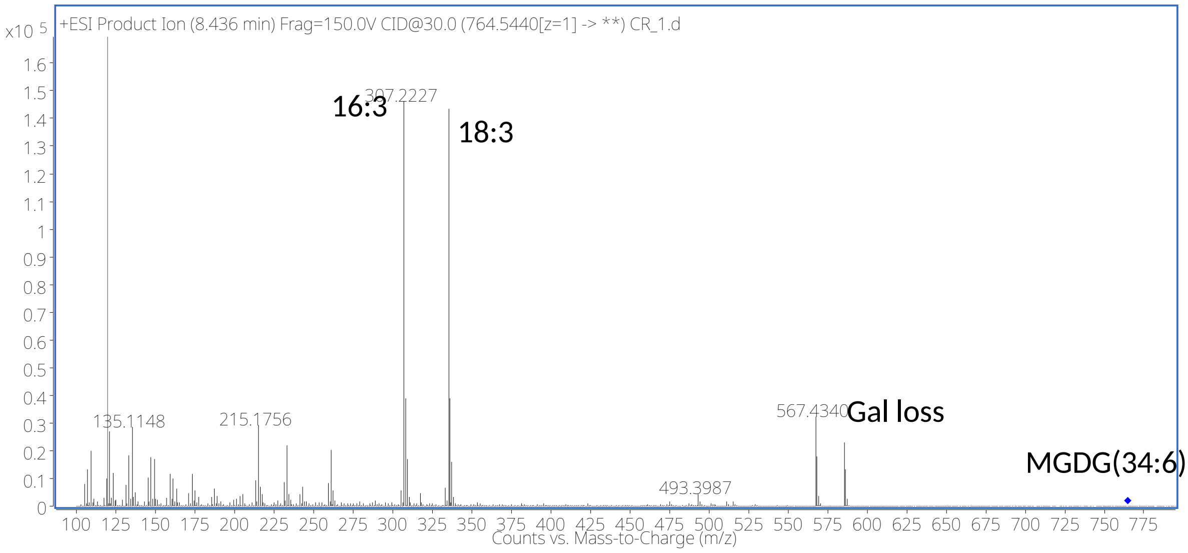

***Chlorella* replete**

MGDG(36:5)

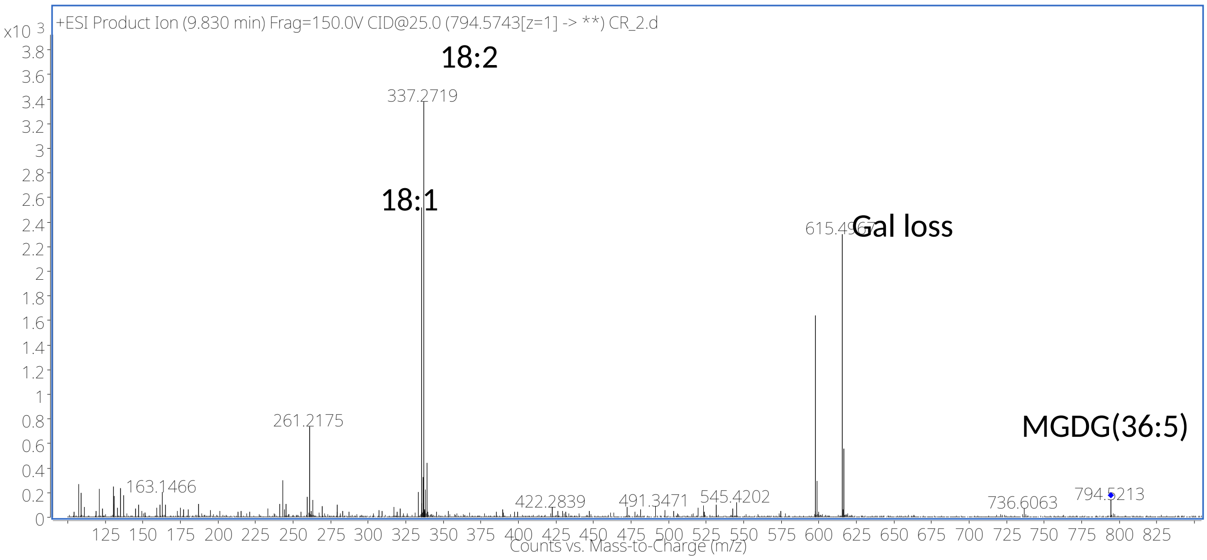

***Chlorella* replete**

MGDG(36:6)

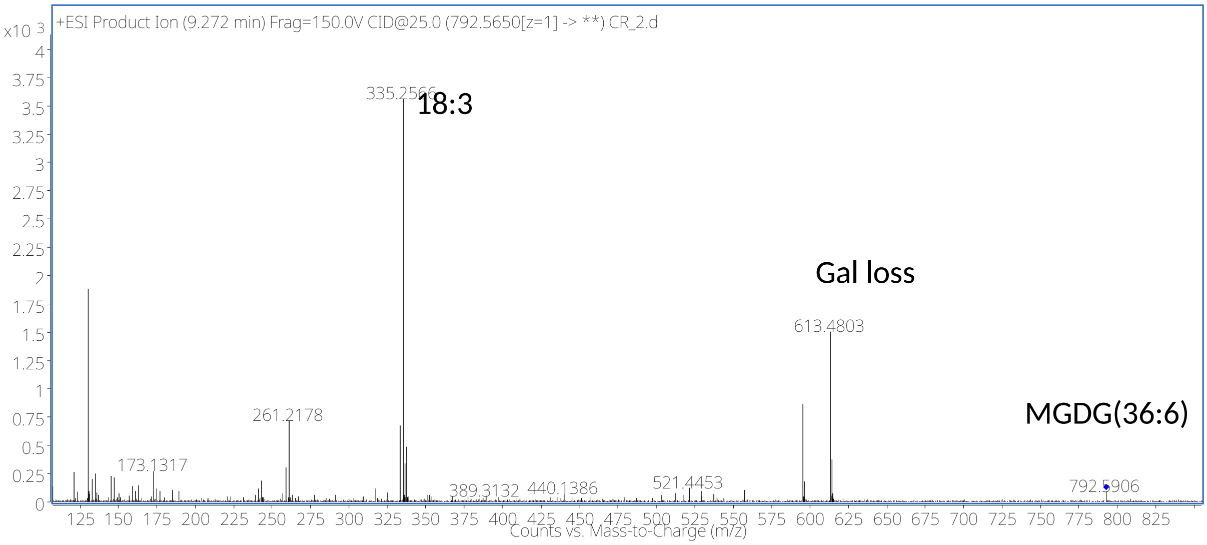

Supplement: Supplementary file 2 [file pone.0103389.s001.docx]
